# Does Cognitive Function Increase over Time in the Healthy Elderly?

**DOI:** 10.1371/journal.pone.0078646

**Published:** 2013-11-11

**Authors:** Jocelyne de Rotrou, Ya-Huei Wu, Jean-Bernard Mabire, Florence Moulin, Laura W. de Jong, Anne-Sophie Rigaud, Olivier Hanon, Jean-Sébastien Vidal

**Affiliations:** 1 Service de Gérontologie, Hôpital Broca, Assistance Publique-Hôpitaux de Paris, Paris, France; 2 Equipe d’Accueil 4468, Université Paris Descartes, Sorbonne Paris Cité, Paris, France; 3 Equipe d’Accueil 4430, Université Paris Ouest, Nanterre, France; 4 Department of Radiology, Leiden University Medical Center, Leiden, The Netherlands; Cardiff University, United Kingdom

## Abstract

**Background:**

In dementia screening, most studies have focused on early cognitive impairment by comparing patients suffering from mild dementia or mild cognitive impairment with normal subjects. Few studies have focused on modifications over time of the cognitive function in the healthy elderly. The objective of the present study was to analyze the cognitive function changes of two different samples, born > 15 years apart.

**Method:**

A first sample of 204 cognitively normal participants was recruited in the memory clinic of Broca hospital between 1991 and 1997. A second sample of 177 cognitively normal participants was recruited in 2008–2009 in the same institution. Both samples were from the same districts of Paris and were assessed with the same neuropsychological test battery. Mean cognitive test scores were compared between 1991 and 2008 samples, between < 80 years old and ≥ 80 years old in 1991 and 2008 samples, and finally between subjects < 80 year old of 1991 sample and subjects ≥ 80 years old of the 2008 sample. Means were compared with T-tests stratified on gender, age-groups and educational level.

**Results:**

Cognitive scores were significantly higher in the 2008 sample. Participants < 80 years old outperformed those ≥ 80 in both samples. However, participants < 80 years old in 1991 sample and subjects ≥ 80 in the 2008 sample, born on average in 1923, performed mostly identically.

**Conclusion:**

This study showed a significant increase of cognitive scores over time. Further, contemporary octogenarians in the later sample performed like septuagenarians in the former sample. These findings might be consistent with the increase in life expectancy and life span in good health. The study highlights the necessity to take into account factors which may contaminate and artificially inflate the age-related differences in favor of younger to the older adults.

## Introduction

In the setting of memory clinics, researchers and clinicians are increasingly interested in diagnosing Alzheimer’s disease and related disorders at a pre-dementia stage [1]. If pharmacological treatments are recognized to be most effective before the irreversible neurodegenerative damage, there is an urgent need to identify the earliest stages of dementia. Early detection of cognitive impairment implies a better knowledge of the healthy elderly and therefore addresses the issue of cognitive function changes in the healthy population over time.

Since the mid-80 s, a worldwide increase of intelligence quotient in young adults known as the Flynn effect (FE) [2], [3] has been reported mainly on fluid intelligence and low cultural factor tests. But, in the context of aging, FE has a larger meaning and has been well documented on tests of episodic memory and executive function [4], [5], [6], such as Trail-Making Test [7], Digit Symbol test [8], and Raven Progressive Matrices [9]. Published studies on the FE in aging, sometimes designated as the Flynn-like effect, have clearly indicated that in developed countries, socio-economic factors constitute one type of factors, interacting with other ones, such as physical and mental health, nutrition, culture, selective immigration-outmigration, new technologies, etc., susceptible to positively or negatively influence individuals’ evolution [5], [6], [10] More recent studies indicate that in higher-income countries, health progress resulting from successful primary prevention and medical treatments particularly regarding vascular risk factors, better education as well, are examples of contributive factors that on the one hand improve cognitive function and on the other hand decrease or delay risk of prevalent dementia [11], [12]. However, improvement of cognitive function addresses the issue of knowing whether increased cognitive scores mean a real increase of cognition or an increase of resources enabling individuals to better challenge cognitive situations, as measured by cognitive tests.

In the elderly, the FE is a broad and complex concept, difficult to disambiguate. Indeed, it remains difficult to fractionate and to quantify with precision the weight of all factors contributing to changes over time in the healthy adults. Thus, FE is a generic concept (such as Memory or Language) allowing, in a practical way, to summarize a set of factors which generate changes over time. The FE might consist of a combination of cohort effect (effects of being born at about the same time, exposed to the same events in society, and influenced by the same demographic trends) and period effect (variation over time periods affecting individuals simultaneously, often resulting from shifts in social, cultural, technological or physical environments) [13], [14].

Increasing cognitive performances in the elderly raise the unanswered question of limits of cognition. Even if this crucial question is beyond the scope of the present study, and has no satisfactory explanation, researchers in the field of aging cannot ignore the existence of a plausible Flynn effect and its potential implications. Within this context, we were interested in the evolution of cognitive function in healthy old participants, using the Cognitive Efficiency Profile (CEP) [15], a validated neuropsychological battery. The objective of the present study was to investigate in two French healthy cohorts cognitive changes over time and bring insight into age, cohort and period effects.

## Materials and Methods

### Samples

The present study is a retrospective study. Data were extracted from two sources.

A first sample of 204 elderly participants was selected between 1991 and 1997 from the patients attending the memory clinic of the Broca hospital, Paris, France. Subjects underwent a comprehensive geriatric assessment, including a neuropsychological assessment (CEP). They were considered to have normal cognition and no major depression. These subjects did not meet the criteria of dementia or pre-dementia according to DSM III-R [16]. From this sample, normative data of the CEP were published in 1997 [17]. Only means and standard deviations from this previous study were available and were used for the present one.

A second sample was composed of 127 elderly attending the memory clinic of the Broca hospital between 2008 and 2009 and 50 caregivers of patients included in the SIGAL study (Study of IGF system on Alzheimer’s disease), registered on Clinical trials.gov Web (number NCT00647478) [18]. They underwent a comprehensive geriatric assessment, including the same neuropsychological assessment (CEP) and were considered to have normal cognition without major depression. For the 2008 sample, subjects did not meet the criteria of dementia according to DSM IV-TR [19] or of MCI according to Petersen [20].

The population attending the memory clinic of Broca hospital came from different districts of Paris. Both samples were extracted from the same parent population in terms of districts. They also were comparable according to common socio demographic characteristics (age, gender). Therefore, both samples were comparable city-dwellers and could be considered representative of aged subjects residing in French large cities.

All data were anonymized prior to the study. All participants gave their consent for being anonymously included in our clinical research database. For the 50 caregivers in the SIGAL study, written informed consents were available. For the present non interventional study, no review from the local institutional review board was needed. Given the retrospective nature of the present study, no written informed consent was available.

### Cognitive Assessment

Each participant had a comprehensive neuropsychological assessment with the CEP. A detailed description of this tool can be found elsewhere [15]. Briefly, the CEP contains the following tests:

- Four items about orientation and autobiographical memory.
- Six sub-tests of the Memory Efficiency Profile of Rey [21]: naming, 3 subtests of visuo-spatial recognition, immediate free recall and delayed free recall of 12 drawings.
- Logical memory with two free recalls of a narrative story.
- Visuo-spatial memory test with the reproduction of a complex figure.
- Association ability and associative memory with cued recall of 12 couples of images: subjects had to use the strategy of association in order to memorize.
- Semantic encoding and free recall of 24 pictures of objects: subjects had to classify 24 pictures into 5 semantic categories (6 bathroom objects, 5 kitchen objects, 6 office objects, 4 clothing items and 3 intruders). A free recall was then required.

All tests were scored with a maximum of 12 points except orientation and autobiographical memory, scored with a maximum of 4 points. A global score with a maximum of 100 points was derived from orientation and autobiographical memory, immediate free recall, delayed free recall, logical memory 1 and 2, visuo-spatial memory, cued recall, semantic encoding and semantic recall. The global cognitive functioning was also assessed using the Mini-Mental State Examination [22].

### Statistical Analysis

Education was categorized into 3 levels: primary school, secondary school and above high school diploma. Age was categorized into two groups: < 80 years old and ≥ 80 years old.

First, general characteristics of the 1991 and 2008 samples were compared using Chi square or T-test for categorical or continuous variables respectively. Cognitive tests scores of the 2 samples were plotted and compared using T-tests. Analysis was repeated after stratification on age group, gender and education using a stratified T-test [23].

Second, each sample was divided by age-groups: participants of the 1991 sample ≥ 80 years old, i.e., born on average in 1905; participants of the 1991 sample < 80 years old, i.e., born on average in 1923; participants of the 2008 sample ≥80 years old, i.e., born on average in 1923; and participants of the 2008-sample < 80 years old, i.e., born on average in 1937. Cognitive tests scores were plotted and compared by age-groups separately for 1991 sample and 2008 sample. Analyses were repeated after stratification on gender and education using a stratified T-test.

Third, the 4 groups were drawn on a single plot and participants from the 2 samples born on average in 1923 (the younger ones from the 1991 sample and the older ones from the 2008 sample) were specifically compared using t-test stratified on age group, gender and education.

Lastly, a sensitivity analysis was conducted after excluding the fifty participants (in the 2008 sample) who were taken from the control group of the SIGAL study, in order to verify that the differences between the 2 samples were not driven by their inclusion. Statistical analyses were performed with R statistical software: the R Development Core Team (2011), Vienna, Austria http://www.R-project.org. In all analyses, the 2 sided alpha-level of 0.05 was used for significance testing.

## Results

### Sociodemographic Characteristics

The 1991-sample was composed of 76.2% (N = 137) women and the sample mean age was 73.2 (Standard Deviation = 10.4). The 2008-sample was composed of 70.1% (N = 124) women and the sample mean age was 73.5 (8.3). Comparison of general characteristics of the 1991 and 2008 samples showed no differences in terms of age or gender (table 1). Meanwhile, educational level was significantly different with lower and higher educational levels in the 1991 sample and more middle educational level in the 2008 sample (p = 0.02).

**Table 1:** General characteristics of the 1991- and 2008-samples.

Characteristics, M (SD)	91-Sample	08-sample	P*	p†
	N = 204	N = 177		
Men, % (N)	23.8 (67)	29.9 (53)	0.62	
Age	73.2 (10.4)	73.5 (8.3)	0.71	
Age ≥ 80, % (N)	27.9 (57)	24.8 (39)	0.59	
Education, % (N)				
Lower	16.6 (34)	11.8 (21)		
Middle	31.9 (65)	45.8 (81)	0.02	
Higher	51.5 (105)	42.4 (75)		
Neuropsychological testing				
MMSE	28.5 (1.7)	28.9 (1.2)	0.007	0.005
CEP global score	73.5 (11.6)	83.2 (5.7)	<0.0001	<0.0001
Naming	12.0 (0.5)	11.9 (0.3)	0.90	0.57
Categorization	11.7 (0.6)	12.0 (0.1)	<0.0001	<0.0001
Cued recall	11.2 (2.0)	11.9 (1.5)	<0.0001	<0.0001
Immediate free recall (IFR)	8.72 (1.85)	10.4 (1.3)	<0.0001	<0.0001
Delayed free recall	8.23 (2.15)	10.1 (1.4)	<0.0001	<0.0001
IFR / Semantic encoding	8.34 (2.1)	9.25 (1.30)	<0.0001	<0.0001
Logical memory 1	6.05 (2.09)	7.05 (1.70)	<0.0001	<0.0001
Logical memory 2	8.46 (2.14)	9.76 (1.34)	<0.0001	<0.0001
Visuo-spatial memory	6.71 (2.30)	8.88 (1.88)	<0.0001	<0.0001
Visuo-spatial recognition	7.99 (3.10)	9.45 (2.68)	<0.0001	<0.0001

* T-test;

† T-test stratified on gender, age and educational level; M (SD), Mean (Standard Deviation); MMSE, Mini-Mental State Examination; CEP, Cognitive Efficiency Profile.

### Performances on CEP

**Figure 1:**
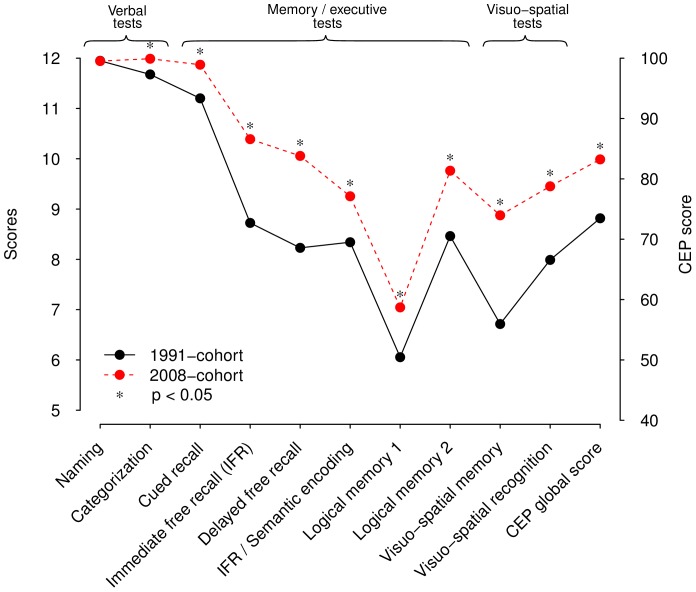
CEP global score and sub-scores of 1991- and 2008-samples.

First, CEP global score and sub-scores were all significantly higher in the 2008 sample with the exception of naming (figure 1). Mini-Mental State Examination was also higher in the 2008 sample compared with the 1991 sample, although the scores were very high and only slightly different (28.5 vs. 28.9). When the analyses were stratified on gender, educational level and age groups, the results remained unchanged except for Mini-Mental State Examination that was no longer significantly different (p = 0.08).

**Figure 2:**
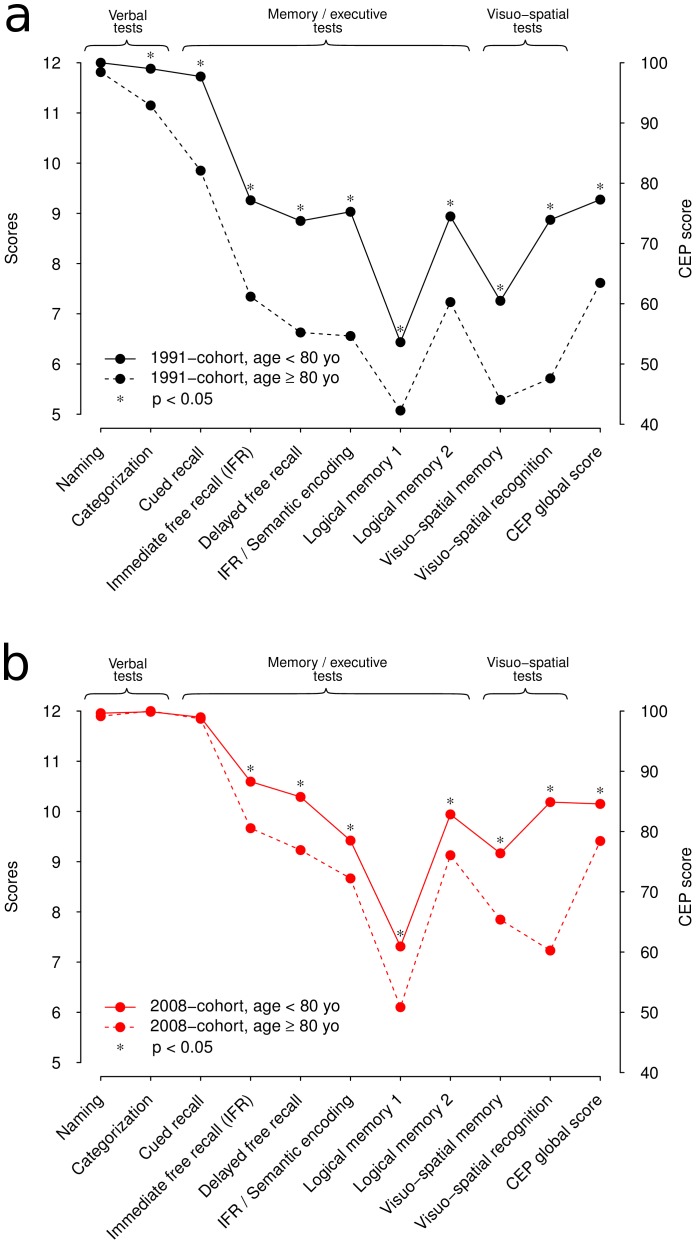
Comparison between participants < 80 yo and participants ≥ 80 yo. in the 1991-sample (a) and in the 2008-sample (b).

Analyses among younger and older participants for the 1991 and 2008 samples taken separately showed significantly higher scores for the younger participants compared with the older ones for both samples (see table 2 and figures 2a and 2b), except for naming in the 1991 sample, and naming, categorization and cued recall for the 2008 sample. When the analyses were stratified on gender and educational level the results remained unchanged.

**Table 2:** Comparison of CEP and sub-scores according to age groups among 1991-and 2008-samples separately.

Neuropsychological testing, M (SD)	1991-ample		p*	p†	2008-sample		p*	p†
	Old	Young			Old	Young		
	N = 57	N = 147			N = 39	N = 138		
MMSE	27.0 (2.2)	29.0 (1.0)	<0.0001	<0.0001	28.5 (1.5)	29.1 (1.0)	0.01	0.03
CEP global score	63.5 (12.9)	77.3 (8.5)	<0.0001	<0.0001	78.4 (5.0)	84.6 (5.18)	<0.0001	<0.0001
Naming	11.8 (0.9)	12.0 (0)	0.009	0.06	11.9 (0.5)	11.9 (0.2)	0.29	0.33
Categorization	11.1 (0.9)	11.9 (0.4)	<0.0001	<0.0001	12.0 (0)	12.0 (0.1)	0.42	0.53
Cued recall	9.85 (3.26)	11.7 (0.7)	<0.0001	<0.0001	11.8 (0.5)	11.9 (0.4)	0.71	0.98
Immediate free recall (IFR)	7.34 (1.88)	9.26 (1.54)	<0.0001	<0.0001	9.67 (1.34)	10.59 (1.28)	0.0001	0.0004
Delayed free recall	6.63 (2.38)	8.85 (1.69)	<0.0001	<0.0001	9.23 (1.33)	10.29 (1.28)	<0.0001	<0.0001
IFR / Semantic encoding	6.56 (2.33)	9.03 (1.6)	<0.0001	<0.0001	8.67 (1.30)	9.42 (1.25)	0.002	0.004
Logical memory 1	5.07 (2.01)	6.44 (2.00)	<0.0001	0.003	6.10 (1.67)	7.31 (1.62)	<0.0001	0.0005
Logical memory 2	7.23 (2.20)	8.94 (1.92)	<0.0001	0.0004	9.13 (1.42)	9.94 (1.26)	0.0006	0.007
Visuo-spatial memory	5.29 (2.18)	7.26 (2.10)	<0.0001	<0.0001	7.85 (1.80)	9.17 (1.81)	<0.0001	0.0008
Visuo-spatial recognition	5.71 (3.21)	8.87 (2.57)	<0.0001	<0.0001	7.23 (2.86)	10.19 (2.17)	<0.0001	<0.0001

* T-test;

† T-tests stratified on gender and educational level; Mean (Standard Deviation); MMSE, Mini-Mental State Examination; CEP, Cognitive Efficiency Profile.

**Figure 3:**
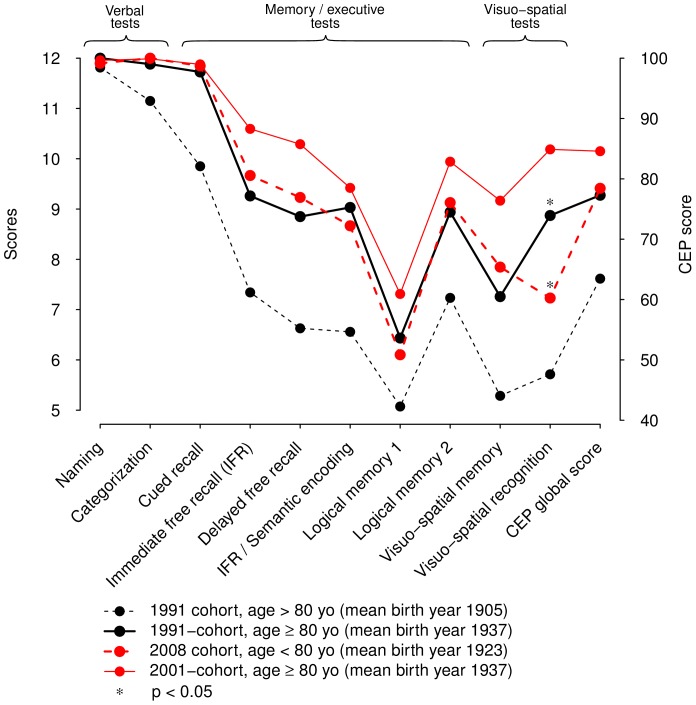
Comparison according to age and birth date in the 1991-and 2008-samples.

Figure 3 shows global score and sub-scores of the CEP in the four groups (i.e., 1991 sample ≥ 80, mean birth year 1905; 1991 sample < 80, mean birth year 1923; 2008 sample ≥ 80, mean birth year 1923; 2008 sample < 80, mean birth year 1937). Participants from the 2 samples born on average in 1923 performed identically on all cognitive tests, except for visuo-spatial recognition (p = 0.002). When analyses were repeated after excluding from the control group participants of the SIGAL study (N = 50), the results remained unchanged for all cognitive measures.

## Discussion

The objective of the present study was to investigate the changes over time in cognitive function of healthy old participants. The study was carried out by analyzing the changes on CEP, a battery of neuropsychological tests, among two different samples assessed 18-years apart. Certainly, there was a difference in the time periods of the neuropsychological assessment as well as between the former and later samples at individuals’ level. But, given that standard socio demographic characteristics of the samples at the level of means and SD were similar, a comparison of their cognitive scores was legitimate.

Results showed that over the 18-year period, there was a significant increase in CEP scores. The 2008 sample scored almost 10 points higher than the 1991 sample. All CEP sub-scores were significantly higher in the 2008 sample compared with the 1991 sample, except one verbal task, the naming task. In this analysis comparing two samples at two different periods of time i.e., in 1991 and in 2008, the changes were composed of a combination of cohort and period effects. Such a combination could be summarized as a Flynn effect in its large meaning, as previously defined. Our findings are concordant with other studies showing that later born cohorts have better cognitive function than do earlier born cohorts [10], [11]. According to our findings which corroborate published data, we believe that the FE in the elderly might not correspond to an increase of intelligence. The FE might only reflect the increase of resources enabling individuals to better cope with challenging cognitive situations.

When samples were divided into two age-groups, participants < 80 years outperformed those ≥80 years in both samples, except for naming task in the 1991 sample and naming, categorization and cued recall tasks in the 2008 sample. Cross sectional analyses often overestimate the impact of brain aging on cognitive decline by confounding age and cohort effects. Indeed, the observed changes might be a combination of age and cohort effects. The cross sectional nature of this analysis precluded us from breaking up those changes between age and cohort effects.

When birth dates of participants were taken into account (figure 3), no significant differences were observed between participants born on average in 1923: < 80 years old of 1991 sample and ≥ 80 years old of 2008 sample, except for visuo-spatial symbols recognition task that seemed to be more sensitive to aging. For this analysis, the changes might be composed of an undividable mix of the age and period effects. However, since the changes were not different from 0, one can assume two different hypotheses, apart from involvement of confounding factors or bias. First, the age and period effects were both unimportant and close to 0. This could suggest that cognitive aging may not be an inescapable deterioration, a constant decline. Some studies indeed suggest that the observed age-related changes might mostly be attributed to Flynn effect (cohort + period effects). Second, between 1991 and 2008 and in those two samples, the change in cognition related to age effect would be counterbalanced by the change in cognition related to the period effect.

The cognitive increase appeared to be task-depending, i.e., more or less strong in all fluid-like tasks tested in our study. This finding reflects differences between fluid and crystallized abilities [24]. In our results, a ceiling effect might explain the overlap of scores on naming, categorization (semantic encoding), or cued recall after using the strategy of association (figures 2B and 3). Nevertheless, another explanation is that specific semantic components involved in these tasks are more resistant over time. Semantic abilities usually refer to over-learned and automated knowledge which are considered to be less sensitive to normal and pathological aging. Naming or classifying objects, for instance, take part in daily life. Given the frequent activation of their underlying neuronal pathways, semantic abilities are strengthened and enable compensations that may delay the clinical onset of most neurodegenerative diseases, or reduce intensity of symptoms.

### Implications of the Study

Our study shows that contemporary octogenarians perform as septuagenarians did eighteen years apart. It could be emphasized that the cut young-old/old-old [25] which was around 60 years old a few decades ago, would be rather around 80–85 nowadays. Baltes’ work demonstrated that at the beginning of the 2000 years, 70-year-olds were comparable to 65-year-olds who lived 30 years ago [26]. Aging is not only a matter of dealing with resource loss. However, successful aging in the healthy elderly has some limits. Comparisons with the oldest individuals called the old-old or fourth age population would probably lead to different findings [26]. With aging, mainly in the fourth age population whose resource deficits are greater, compensation factors will become saturated and will reach a critical level. Beyond a certain age, or below a critical level, although variable within individuals, an age-related decline will overweight all other factors. However, it is worthy of mentioning that more and more studies comparing cohorts older than 90 years with younger ones also demonstrate cognitive increase associated with higher scores in activities of daily living as well as a lower risk of prevalent dementia in more recently born populations compared with earlier born ones [11], [12].

Since aged individuals have increased cognitive scores today than before, updating normative data of tests in the context of dementia screening is necessary. Updated norms reduce the risk of false negative cases and consequently the risk of postponing management. Analysis of our database indeed indicates that the former normative data do not allow any further reliable interpretation of results on most tests of the CEP. An average score considered as normal with the former standards (1991 sample) will be considered as insufficient or even pathological with the new norms (2008 sample). All tests may not have become outdated, but their norms can be outdated. Updating norms mainly concerns the “fluid” components of the cognitive function. However, regarding technological changes, updating may be insufficient. The selection of activities assessed by batteries of tests has to be changed over time. Researchers and clinicians have also to deal with the need of designing new tests more adapted to newer old participants.

### Limits of the Study

Some caveats however are to be considered when interpreting the results. First, some specific executive functions, usually considered being sensitive to aging, such as double tasks or reaction-time tests, or timed tests were not measured and one could assume larger differences between age groups with those tests. Second, our data could not been analyzed using Age-Period-Cohort (APC) model [5] because we only had 2 points of assessment (1991 and 2008). Thus we could not determine the weight of the different age-period-cohort effects on the observed changes. Third, given the actual use of more rigorous diagnostic approaches in the screening of patients suffering from dementia, some patients with mild cognitive impairment (MCI) according to recently revised criteria may have been included in the 1991-sample. However, even before the emergence of the MCI concept, patients scoring between normal and pathological were called “borderline cases” in our practice [15] and were not included in research protocols on healthy adults. Another limitation could be that in both samples, results involved the surviving participants. This could be a bias which reduces the age-effects per se. Individuals who participate and are followed until the end of longitudinal studies are the most resistant to deleterious biological and environmental factors and those who benefit the most from adaptive capacities [27], [28]. Lastly, the two samples from 1991 and 2008 were composed of different individuals and one could hypothesize that the higher cognitive performances in the 2008 sample were driven by educational level differences, although in the 2008 sample there were less higher educational levels and stratification on educational level did not modify the results. It also could be argued that the representativeness of samples might be questionable since the participants were recruited in a memory clinic. However, both samples were extracted from the same parent population and had comparable socio-demographic characteristics.

### Strength of the Study

Most published studies on cognitive aging compare older participants with younger participants and usually present data in terms of age-related decline. Few studies assessed normal cognitive aging in two samples using the same neuropsychological battery of tests > 15 years apart. Having highlighted increase of cognitive scores over time, our study has challenged the assumption that cognitive aging means inexorable decline. Indeed, what is usually called age-related decline is not a 100% age-related decline. The decline may be due partly to age (particularly in the fourth age population), partly to differences between generations, lifestyle, socio-economic status, culture, medical and personal factors. Even if it remains challenging to precisely fractionate the global pattern of cognitive increase into its different contributive factors, future studies should attempt to clarify. However, in clinical practice, taking into account the cumulative effect of these different factors can help discriminating normal aging from pathology. In the future, in order to better understand cognitive changes over time, either in terms of decrements or increments, it could be relevant not only to adjust scores for age, educational level, and gender as usual, but also to control birth-dates.

## Conclusion

In this study, increased cognitive scores in more recently born healthy elderly compared with those born >15 years before, were observed. This finding corroborates results from other recent studies on cohort differences involving larger sample sizes [11], [12]. This increase is consistent with the increase in life expectancy and lifespan in good health. The cognitive increase is observed in fluid-like tasks and corresponds to a complex combination of age-period-cohort effects. According to our results and in our present knowledge, increased cognitive scores do not systematically correspond to an increase of intelligence. Increased resources enabling to better challenge cognitive situations might facilitate adaptation to cognitive situations as measured by cognitive tests. Whatever, the need of improving early diagnosis of dementia, urges caution when assessing the cognitive function of aged participants. A better knowledge of the healthy elderly is helpful in the setting of dementia screening. Aging is a multi-factorial process with modifiable and non-modifiable risk factors. Improvement of modifiable risk factors, intellectual stimulation and new technologies use are, to date, effective means to achieve healthy aging.
